# Perampanel and childhood absence epilepsy: A real life experience

**DOI:** 10.3389/fneur.2022.952900

**Published:** 2022-08-11

**Authors:** Francesca Felicia Operto, Alessandro Orsini, Gianpiero Sica, Chiara Scuoppo, Chiara Padovano, Valentina Vivenzio, Valeria de Simone, Rosetta Rinaldi, Gilda Belfiore, Roberta Mazza, Salvatore Aiello, Luigi Vetri, Serena Donadio, Angelo Labate, Grazia Maria Giovanna Pastorino

**Affiliations:** ^1^Child and Adolescent Neuropsychiatry Unit, Department of Medicine, Surgery and Dentistry, University of Salerno, Salerno, Italy; ^2^Pediatric Neurology, Pediatric Department, Azienda Ospedaliero-Universitaria Pisana, Pisa, Italy; ^3^Azienda Sanitaria Locale Salerno, Salerno, Italy; ^4^OASI Research Institute- IRCCS, Troina, Italy; ^5^Department of Psychology, Educational and Science and Human Movement, University of Palermo, Palermo, Italy; ^6^Department of Biomedical, Dental Sciences and Morphological and Functional Images, University of Messina, Messina, Italy

**Keywords:** perampanel, childhood absence epilepsy, efficacy, tolerability, children

## Abstract

**Objectives:**

The aim of our study was to evaluate the effectiveness and tolerability of perampanel (PER) as first add-on and as second line monotherapy in subjects with childhood absence epilepsy.

**Methods:**

Our sample consisted of 20 patients with childhood absence epilepsy, aged between 8 and 10, already in therapy with a first antiseizure medication with incomplete seizure control. PER was added as first add-on in a dose ranging from 3 to 8 mg/die with 1- 2 mg/week increments. The patients that were seizure-free were shifted to a PER monotherapy. All patients underwent a standardized neuropsychological evaluation in order to assess non-verbal intelligence and executive functions before adding PER and after 6 months of drug therapy. All parents completed two questionnaires, in order to assess the emotional-behavioral problems and parental stress.

**Results:**

15/20 patients responded to add-on PER and were seizure-free, in 3/20 patients we observed a reduction of seizure frequency <50%, and in the 2 remaining patients the add-on therapy with PER did not lead to a reduction in seizures frequency from baseline. The patients who were seizure-free were switched to PER monotherapy. 9/15 patients remained seizure-free in monotherapy with PER. In the first month of therapy with PER 2/20 patients (10%) reported mild, transient side effects of irritability, headache and dizziness, which did not lead to discontinuation of therapy. Adjunctive treatment with PER did not negatively affect non-verbal intelligence, executive functions, emotional/behavioral symptoms of children and parental stress levels.

**Significance:**

Our clinical experience in real life showed that PER appears to be effective in the control of absence seizures in childhood absence epilepsy, with a favorable tolerability profile. PER would seem effective on absence seizures even in monotherapy. Further studies with larger samples, longer follow-up and controlled vs. placebo (or other first choice antiseizure medications) are needed to confirm our data.

## Key points

- We evaluated the effectiveness and tolerability of perampanel as first add-on and as monotherapy in 20 patients with childhood absence epilepsy.
- 15/20 (75%) patients were seizure-free with add-on therapy, 9/15 (60%) patients remained seizure-free in monotherapy with perampanel.
- 2/20 patients (10%) reported mild, transient side effects in the first months which did not lead to discontinuation of therapy.
- Perampanel did not negatively affect non-verbal intelligence, executive functions, emotional/behavioral symptoms and parental stress.
- Our real life experience showed that perampanel seems effective in the control of absence seizures, with a favorable tolerability profile.

## Introduction

Childhood absence epilepsy (CAE) is a well-known and common pediatric epilepsy syndrome affecting 10–17% of all children with epilepsy (1). Seizures usually begin between 4 and 10 years of age, with a peak around 6–7 years, in a previously healthy and typically developing child. CAE occurs more often in girls than in boys (2). Seizures occur many times daily and consist of brief staring spells, sometimes with rhythmic eye blinking or motor automatisms, lasting seconds, with immediate return to the baseline level of awareness and activity. On electroencephalography (EEG), seizures are characterized by a highly recognizable pattern of generalized (bilateral, symmetric and synchronous) 3 Hz spike and wave discharges (3). Childhood absence epilepsy is often thought of as a benign, self-limited epilepsy, but there are significant cognitive, behavioral and psychiatric comorbidities that must be detected early and addressed separately. Baseline rates of inattention are 30–40% and do not improve with successful treatment of seizures (4). One quarter may have subtle cognitive or language impairments, and more than half are found to have psychiatric diagnoses when formally assessed, particularly attention deficit hyperactivity disorder, anxiety and emotional problems (5).

Three antiepileptic medications have been commonly used as first-choice agents for childhood absence epilepsy-ethosuximide (ETX), valproic acid (VPA), and lamotrigine (LTG) (6)-alone or in combination. VPA and LTG are also effective treatments for many patients, but when compared to ETS, VPA has more adverse effects and LTG is less effective (3). Not all patients with childhood absence epilepsy will become seizure free on the first or second medication. Considering that drug-resistant childhood absence epilepsy can occur in some patients, and that VPA should be avoided in female of childbearing potential, it would be useful to evaluate the efficacy and tolerability of other antiseizure medications. Among the new antiseizure medications, Perampanel appears to be a promising therapeutic alternative.

Perampanel (PER) is a third generation antiseizure medication (ASM) based on a different mechanism of action compared to the previous one, performing as a selective non-competitive antagonist of the glutamate AMPA receptor ion channel (7). PER was approved as adjunctive treatment of focal seizure in patients aged ≥4 years (and as monotherapy in the USA), and as adjunctive treatment of primary generalized tonic-clonic (GTC) seizure associated with idiopathic generalized epilepsy (IGE) in patients aged ≥ 12 years (and ≥7 years in the EU) (8, 9). Available studies on the efficacy and safety of PER in adolescents have shown an overall favorable risk-benefit profile, with generally mild or moderate adverse events (10–12).

The most recent literature evidence suggested that PER can be considered as a broad spectrum antiseizure medication, but the effectiveness and tolerability of PER in the different type of generalized seizure had not been systematically assessed. The efficacy of PER on absence seizures was performed through a *post-hoc* analysis in patients who participate in a randomized controlled trial (study 332) and open label extension studies (13, 14). Outcome for absences seizures are reported for 27 patients in treatment with PER and 33 placebo controls; seizures freedom was reported in 22.2% of patients receiving PER vs. 12.1% of the controls and increased absence seizure frequency was observed in 29.6 vs. 45.5% of patients, respectively (13).

However, outcomes with PER are reported in people with absences epilepsy syndromes in two observational studies (14, 15). In 37 patients aged ≥12 years (10 with childhood absence epilepsies, 21 with juvenile absence epilepsies and 6 with adult-onset absence epilepsy) the authors observed that the seizure frequency was reduced by 71.4% from baseline at 1 year after PER addition, and 51.4% of patients were seizure-free after 12 months of +follow-up (14).

In a systematic review of Trinka et al. (16) the authors analyzed the data of epileptic patients with different form of generalized seizures, among which 112 with absences seizures (absence childhood epilepsy = 43), extrapolated from one randomized controlled trial (study 332), nine observational studies and two cases studies. The authors concluded that adjunctive PER was useful in the treatment of absence seizures in the context of IGE, despite the low sample size involved a low statistical power. In fact, the size of the treatment effect was generally smaller than that observed for myoclonic seizures, but estimates were based on fewer patients. Moreover, another important consideration that emerged from this study was that there was no evidence of any association between PER and seizure aggravation in generalized epilepsies. Finally, the tolerability profile of adjunctive PER in generalized seizures was consistent with that reported in the randomized controlled trials of adjunctive PER in patients with focal seizures. The most frequently reported side effects were somnolence, dizziness and irritability and the retention rate reported for generalized seizures was >70% across the different studies, suggesting that adjunctive PER was overall well tolerated.

To the best of our knowledge, there are no studies analyzing the effect of adjunctive PER and PER monotherapy in patients with childhood absence epilepsy.

The aim of our study is to evaluate the effectiveness and tolerability of PER as the first add-on and in second line monotherapy in children with absence seizures in a real life experience.

## Methods

### Study design

Our study was a single center, retrospective, observational study that aimed to assess the safety and effectiveness of PER in children with childhood absence epilepsy in a real world setting. Patients referred to the Child Neuropsychiatry Unit of the University of Salerno from October 2018 to September 2021.

Inclusion criteria were: (i) a diagnosis of childhood absence epilepsy (ii) the addition of PER as first add-on for the lack of seizure control with a previous antiseizure medication. (iii) absence of neurological (headache, cerebral palsy, neurodegenerative diseases etc.) or psychiatric comorbidities (intellectual disability, attention deficit/hyperactivity disorder, specific learning disorder, anxiety, depression and psychosis based on DSM-5 (17)) and other relevant medical conditions (endocrinopathies, metabolic, hepatic, cardiac or renal disorders) which could negatively affect neuropsychological performances.

The diagnosis was made by a child neuropsychiatrist with decades of experience in the treatment of epilepsy, on the basis of clinical history, clinical manifestations of seizures and video-EEG characteristics (occurrence of typical generalized spike-and-wave discharges of 3 cycles per second) according with the International League Against Epilepsy classification (18).

Video-EEG recording was performed for 1 h, in wakefulness and with activation tests (hyperpnea and intermittent light stimulation). Perampanel doses administered were determined by the treating physician for each patient in order to achieve optimal seizure control and avoid adverse effects. We carried out the therapeutic drug monitoring of PER based on a HPLC-UV/FL double detection approach, and using ketoprofen as internal standard (19).

As our habitual clinical practice, all the patients underwent a standardized neuropsychological evaluation in order to assess non-verbal intelligence (Raven Progressive Matrices), and executive functions (EpiTrack Junior test) before adding PER (T0) and after 6months of drug therapy (T1). At the same time all the parents completed a self-report questionnaire, in order to assess the emotional-behavioral profile (Child Behavior Checklist) of their children and parental stress (Parental Stress Index). The following factors were considered in our analysis: age, sex, seizure frequency, seizure outcome, concomitant ASMs, PER dose, video-EEG recording, neurological examination, neuroradiological imaging, routine blood analysis.

All parents kept a diary of seizures and of adverse events, as our habitual clinical practice. The efficacy of PER treatment was measured considering the responder rate (at least 50% reduction in seizure frequency); tolerability was evaluated considering adverse events reported by parents.

The aims and procedures of the study were explained to all participants, and written informed consent from the parents was obtained. The study was performed according to the rules of good clinical practice of the Helsinki Declaration and approved by the local Ethics Committee (protocol number 0031994, date of approval March 18, 2020).

### Raven progressive matrices (RPM)

The Raven Progressive Matrices (20) is a test typically used for measuring non-verbal intelligence in subjects aged between 5 and adulthood (21). They are available in different forms.

All participants in our study were administered the Standard Progressive Matrices, that includes five series of 12 elements, which require an increasing cognitive capacity to encode and analyze non-verbal visuospatial information.

Raw scores have been converted in percentiles and age-weighted standard scores with mean = 100 and standard deviation = 15. Scores ≥5° percentile or ≥70 standard score are considered in the norm.

### EpiTrack Junior

EpiTrack Junior is a screening tool for executive functions which is especially sensitive to drug effects and therefore particularly indicated for monitoring ongoing treatment (22). It consists of six subtests (inhibition, visual-motor speed, mental flexibility, visual motor planning, verbal fluency and working memory) that contribute to determining an age-corrected total score. The maximum age-corrected total score is 49. A total score below 32 points indicates an executive functions impairment, according to the following: 29–31 points = mild impairment; ≤28 points = significant impairment. Significant change in two subsequent measures is indicated by a gain of >3 points and a loss of >2 points.

### Child Behavior-Checklist 6-18

The Child Behavior-Checklist 6-18 (CBCL) (23) is a standardized questionnaire for parents that evaluates emotional, social, and behavioral problems in children aged between 6 and 18. The questionnaire consists of 113 questions, to which parents can answer with a Likert scale ranging from 0 to 2 (0 = Not True, 1 = Somewhat or Sometimes True, 2 = Very True or Often True). Raw scores are converted to T-scores, weighted by sex and age. It is possible to obtain the scores of three main scales (“Internalizing Problems,” “Externalizing Problems” and “Total Problems”), six scales based on the DSM-IV (“Affective Problems,” “Anxiety Problems,” “Somatic Problems,” “ADHD Problems,” “Oppositional Defiant Problems,” “Conduct Problems”), and eight empirically based syndrome scales (“Anxious/Depressed,” “Withdrawn/Depressed,” “Somatic Complaints,” “Social Problems,” “Attention Problems,” “Rule-Breaking Behavior,” “Aggressive Behavior”) in which a T-score ≤64 indicates non-clinical symptoms, a T-score between 65 and 69 indicates a borderline range, and a T-score ≥70 indicates clinical symptoms.

### Parenting Stress Index

The Parenting Stress Index (PSI) (24) is a standardized questionnaire for parents that measures the level of stress in the dyad parent-child. The short form of PSI consists of 36 items, to which parents attribute a score on a Likert scale ranging from “5 = strongly agree” to “1 = strongly disagree.”

This self-report is organized in different subscales: Parental Distress (PD), Parent-Child Dysfunctional Interaction (P-CDI), Difficult Child (DC) which evaluate the level of distress a caregiver is experiencing in his/her parental role, the satisfaction in the relationship with their own child, and, lastly, how difficult the management of the child is perceived to be.

The test also allows to evaluate a Total Stress scale (TS). The TS is obtained by adding the relative scores of the three subscales PD, PCD-I and DC.

Raw scores are converted in age-weighted scores. A higher score suggests a higher stress level and a score above 85 indicates clinically significant parental stress.

### Statistical analysis

All neuropsychological scores were expressed as mean ± standard deviation (SD). In order to verify the data distribution, the Kolmogorov-Smirnov normality test was preliminarily performed. Because of the presence of some data not normally distributed, non-parametric methods were employed for our analysis. The mean scores comparison was performed using the Wilcoxon signed-rank test (paired samples). All data were analyzed using Statistical Package for Social Science software, version 23.0 (IBM Corp, 2015).

## Results

### Efficacy and safety of adjunctive PER and PER monotherapy

Our sample consisted of 20 patients with childhood absence epilepsy, aged between 8 and 10 years (males = 7, mean age = 9.25 ± 0.85). At baseline all patients were already in therapy with a first antiseizure medication (4–16 weeks), with incomplete seizure control. None of the patients had generalized tonic-clonic seizures. Seven patients were taking ETS, 5 patients VPA, 5 patients were in therapy with LEV, and the remaining 3 with LTG. The seizure frequency was multi-daily in all patients (mean = 21 ± 7.36/die). PER was added to all patients as first add-on in a dose ranging from 3 to 8 mg/die (mean dose = 4.8 ± 1.32/die) with 1–2 mg/week increments, reaching the full dose in 2–4 weeks.

In the first month of therapy with PER 2/20 patients (10%) reported mild side effects of irritability, headache and dizziness during the first month of PER treatment, however transient and which did not lead to discontinuation of therapy. Other important adverse effects were not reported for the entire duration of PER treatment (mean follow-up = 10.15 ± 4.77 months). Blood tests repeated after 3–6 and 12 months were normal.

15/20 patients responded to the add-on therapy with PER and were seizure-free (5 of them were in therapy with LEV−100%, 5 with VPA−100%, 3 with ETS−43%, and 2 with LTG−67%), in 3/20 patients we observed a reduction of seizure frequency <50% (2 of them were in therapy with ETS and 1 with LTG), and in the 2 remaining patients the add-on therapy with PER did not lead to a reduction in seizures frequency from baseline (both in therapy with ETS). The patient that were not seizure-freewere shifted to another antiseizure medication. In the 15 patients that were seizure-free after PER therapy video-EEG was negative for epileptic abnormalities.

The retention rate after 12 months of follow-up was 75%.

The patients who were seizure-free with adjunctive PER were switched to PER monotherapy (mean follow-up on PER monotherapy = 5.56 ± 1.59 months). 9/15 patients remained seizure-free in monotherapy with PER, in the remaining 6/15 there was a reappearance of absence seizure for which the double antiepileptic therapy was restored with complete seizure control. Table 1 summarizes the main clinical characteristics of all patients and the response to PER.

**Table 1:** Sample clinical and demographic characteristics.

**Pt** **n°**	**Sex**	**Age (years)**	**Epilepsy duration (weeks)**	**Seizure frequency (for days)**	**1°ASM**	**PER** **dose (mg** **/day)**	**Seizure control with PER**	**PER** **monotherapy**	**Total follow-up (months)**	**Follow-up in mono with PER (months)**	**AEs with PER**
1	F	10	4	20	ETS	4	Yes	Yes	16	8	
2	M	10	12	30	VPA	4	Yes	Yes	18	6	
3	F	8	5	15	LEV	3	Yes	Yes	13	4	
4	F	8	10	30	ETS	6	No	No	3	-	
5	M	10	8	20	VPA	4	Yes	Yes	13	8	
6	F	9	7	15	LTG	6	No	No	3	-	
7	F	8	12	20	ETS	3	Yes	No	14	-	
8	M	10	9	15	VPA	4	Yes	No	12	-	
9	F	9	6	30	LEV	4	Yes	Yes	9	4	IR
10	M	10	8	40	LTG	6	Yes	No	10	-	
11	F	10	10	20	LEV	8	Yes	No	12	-	
12	M	10	16	15	VPA	6	Yes	No	8	-	
13	F	9	4	20	LEV	4	Yes	Yes	12	6	DZ, HD
14	M	8	6	30	VPA	6	Yes	Yes	12	5	
15	M	9	11	15	LTG	6	Yes	No	14	-	
16	F	9	12	20	LEV	4	Yes	Yes	11	5	
17	F	10	12	10	ETS	4	Yes	Yes	14	4	
18	F	10	8	20	ETS	6	No	No	3	-	
19	F	8	10	15	ETS	4	No	No	3	-	
20	F	10	6	20	ETS	4	No	No	3	-	
Mean	M = 7	9.25	8.80	21		4.8	Yes = 15	Yes = 9	10.15	5.56	
SD		0.85	3.17	7.36		1.32			4.77	1.59	

*ASM, antiseizure medication; PER, perampanel; AEs, adverse events; F, female; M, male; ETS, ethosuximide; VPA, valproic acid; LEV, Levetiracetam; LTG, lamotrigine; IR, irritability; DZ, dizziness; HD, headhache; SD, standard deviation*.

### Neuropsychological evaluation

As in our usual clinical practice, we performed a standardized neuropsychological assessment at baseline (before the introduction of PER) and 6 months after the introduction of therapy with PER in add-on. Comparison of mean scores showed that there was no statistically significant difference in Raven's Progressive Matrices, Epitrack Junior, Child Behavior CheckList, and Parental Stress scores after 6 months PER add-on therapy. Table 2 summarizes the mean scores obtained on the neuropsychological tests at time 0 and time 1 and the results of the statistical comparison.

**Table 2:** Statistical comparison of mean neuropsychological scores (Raven's Progressive Matrices, Epitrack Junior, Child Behavior CheckList, and Parental Stress) pre and after 6 months of PER add-on therapy.

**Standardized neuropsychological test**	**Time 0** **(Mean ± SD)**	**Time 1** **(Mean ± SD)**	**Statistic (Wilcoxon Test)**	***p*-value**
**Raven progressive matrices**	98.27 ± 8.97	98.40 ± 8.28	Z = −0.540	*P* = 0.589
**EpiTrack junior**	29.00 ± 6.54	29.73 ± 6.97	Z = −1.132	*P* = 0.258
**Parental Stress index (PSI)**				
Parental distress (PD)	68.87 ± 13.91	69.67 ± 11.57	Z = −0.226	*P* = 0.821
Parent–child difficult interaction (*P*-CDI)	64.33 ± 11.93	65.67 ± 10.50	Z = −0.803	*P* = 0.422
Difficult child (DC)	69.33 ± 15.80	70.33 ± 11.25	Z = −0.122	*P* = 0.903
Total stress (TS)	65.33 ± 12.32	66.00 ± 13.78	Z = −0.142	*P* = 0.887
**Child behavior checklist (CBCL) 6–18 years**				
Anxiety/Depression	56.00 ± 4.41	57.07 ± 7.13	Z = −0.472	*P* = 0.637
Withdrawal/Depression	60.80 ± 7.36	62.47 ± 9.30	Z = −0.971	*P* = 0.332
Somatic complaints	62.27 ± 7.83	61.13 ± 8.91	Z = −1.795	*P* = 0.073
Socialization	60.40 ± 7.10	61.33 ± 10.75	Z = −1.178	*P* = 0.859
Thought problems	56.67 ± 8.34	55.00 ± 6.31	Z = −0.238	*P* = 0.812
Attention problems	59.53 ± 7.05	60.93 ± 6.95	Z = −1.293	*P* = 0.196
Rule-breaking behavior	54.13 ± 5.00	55.20 ± 5.65	Z = −0.510	*P* = 0.610
Aggressive behavior	55.47 ± 5.24	56.60 ± 5.74	Z = −0.567	*P* = 0.571
Affective problems	60.87 ± 8.06	61.53 ± 6.89	Z = −0.541	*P* = 0.589
Anxiety problems	61.27 ± 7.68	61.80 ± 9.84	Z = −0.489	*P* = 0.624
Somatic problems	57.93 ± 6.53	58.67 ± 5.94	Z = −0.313	*P* = 0.754
Attention deficit/Hyperactivity disorder	58.40 ± 6.13	58.67 ± 6.08	Z = −0.600	*P* = 0.549
Oppositional-defiantproblems	56.33 ± 5.38	56.60 ± 4.79	Z = −0.597	*P* = 0.550
Conduct problems	54.87 ± 5.67	55.67 ± 4.72	Z = −0.970	*P* = 0.332
Internalizing problems	55.13 ± 7.81	56.07 ± 6.52	Z = −0.409	*P* = 0.682
Externalizing problems	58.20 ± 8.22	59.13 ± 11.81	Z = −0.118	*P* = 0.906
Total Problem	56.93 ± 8.27	57.40 ± 6.05	Z = −0.313	*P* = 0.755

## Discussion

To the best of our knowledge, this is the first work that analyzes the effectiveness and tolerability of PER as a first add-on and as a monotherapy in patients with childhood absence epilepsy.

Our retrospective study investigated 20 patients aged between 8 and 10 years (males = 7, mean age = 9.25 ± 0.85) diagnosed with childhood absence epilepsy in which PER was added as the first add-on medication for incomplete seizure control.

According to the most recent studies, PER can be considered among the “broad spectrum” antiseizure medication as some evidence suggests its efficacy in reducing both focal and primary and secondary generalized seizures (17). Furthermore, no evidence was found to suspect an association between PER and seizure worsening in generalized epilepsies (17).

Overall, our real-life experience showed a good effectiveness of PER in reducing absence seizure frequency; 15/20 patients (75%) were seizure-free after the introduction of PER. Of the remaining 5 patients, 3 patients achieved a seizure reduction <50% (12%) and 2 patients were not responder (8%), so they were switched to another antiseizure medication. In the 15 patients that were seizure-free after PER therapy video-EEG was negative for epileptic abnormalities. The retention rate after 12 months of follow-up was 75%. Our findings confirm and support data from previous studies, which had already suggested a global absence seizure reduction effect in several forms of epilepsy including childhood absence epilepsy (17). Outcomes for absence seizures are reported in a *post-hoc* analysis of a randomized-controlled trial (PER = 27 vs. placebo = 33), in nine observational studies and in two case studies. In the *post-hoc* analysis of the randomized-controlled trial (13), freedom from absence seizures was reported in 22.2% of patients (6/27) in the PER group and 12.1% (4/33) in the placebo group, and increased absence seizure frequency was observed in 29.6% (8/27) patient that assumed PER and 45.5% (15/33) patients taking placebo (13). Regarding specifically childhood absences epilepsy, outcomes with PER are reported in 43 patients in two observational studies (15, 16). The largest cohort was 37 patients aged ≥12 years (*n* = 21 with juvenile absence epilepsy, *n* = 10 with childhood absence epilepsy and *n* = 6 with adult-onset absence epilepsy) (15). In these 37 patients, primary generalized tonic-clonic seizures frequency was reduced by 71.4% from baseline after 1 year or PER therapy, and 51.4% of patients (19/37) were seizure-free at 12 months and after a visit at the sixth month (67.9% free of primary generalized tonic-clonic seizures, 33.3% free of myoclonic seizures, 48.4% free of absence seizures) (15).

Regarding the tolerability of PER, our data showed an overall good tolerability profile: 2/20 patients (10%) reported mild adverse events characterized by irritability, headache and dizziness during the first month of PER treatment, however these effects were transient and did not lead to a discontinuation of therapy. Other important adverse effects were not reported for the entire duration of PER treatment (mean follow-up = 10.15 ± 4.77 months). Blood tests repeated after 3–6 and 12 months were normal in all patients. The standardized neuropsychological assessment showed that after 6 months of treatment with PER there were no significant changes in the non-verbal cognitive profile. Executive functions (working memory, shifting, inhibition, sustained attention) also did not appear to change from baseline, confirming the results of some previous studies. The impact of PER on cognition was previously explored by several studies and a recent systematic review by Witt & Helmstaedter preliminarily indicates a neutral cognitive profile of PER with no systematic cognitive deteriorations or improvements (21). Our results confirmed those of previous studies, which had shown good tolerability on the cognitive profile in epileptic children receiving PER therapy (25–28).It is important to underline this result since, especially in the pediatric age, cognitive and executive functions are fundamental in terms of good social adaptation, academic performance and good quality of life (29–33).

Concerning the behavioral and emotional aspects, it is known that children with epilepsy are at greater risk of developing internalizing and externalizing problems compared to their peers and these symptoms can get worse with antiseizure medications. Some previous studies highlighted an increase in externalizing symptoms in patients that assumed PER (34–36).

For this purpose, a standardized evaluation of emotional and behavioral problems was made in our sample through a questionnaire administered to parents. As reported by the parents, the additional treatment with PER did not determine in our patients an increase in both internalizing and externalizing problems, confirming data of our previous research (11, 12).

To our knowledge, there are no other previous studies that specifically evaluated stress changes in parents of epileptic children taking PER, but in previous research we found that high level of parental stress was found in parents of children with epilepsy, even in the mildest forms (37–39). In our work, we did not find significant changes in stress levels perceived by parents after the addition of PER.

The patients who were seizure-free with the therapy in add-on with PER, were switched to PER monotherapy (mean follow-up on PER monotherapy = 5.56 ± 1.59 months). 9/15 patients remained seizure-free in monotherapy with PER, in the remaining 6/15 there was a reappearance of absence seizure for which the double antiepileptic therapy was restored, with complete seizure control.

Our data show that PER is effective in controlling absence seizures even in monotherapy.

To the best of our knowledge there are no previous studies that specifically evaluate the efficacy of PER in childhood absence epilepsies, however PER demonstrated good effectiveness and a good safety profile when used as primary therapy or conversion to monotherapy at relatively low doses, in a clinical setting with adult patients with focal seizures and generalized tonic-clonic seizures (40). Real-world data of PER monotherapy in treatment-naïve patients (≥15 years) with focal onset seizures demonstrated good effectiveness and a good safety profile at relatively low doses (41, 42).

In a recent review by Toledano and Gil-Nagel (43) the authors evaluated outcomes of two retrospective multicentre studies in which PER was used as monotherapy; the study shows that low doses (6–8 mg/day) of PER were effective and well tolerated in a subgroup of patients with less severe epilepsies than patients who participated in clinical trials (where PER was used as add-on therapy). In these studies, the retention rate exceeded 90% at 3 months, and 70% at 6, and 12 months. The responder rate was > 75% at 3 months, and the rate of seizure-free patients exceeded 50% at 3 and 6 months, and 37% at 12 months. Compared to other observational studies and clinical trials where PER was used as add-on therapy, no adverse effects other than those already known were observed (44). The authors concluded that in routine clinical practice, conversion to PER monotherapy, at relatively low doses, seem to been an effective and well-tolerated treatment for patients with focal and generalized tonic-clonic seizures.

The main limitations of our study are the low sample size and the retrospective, observational design of the study. Randomized, controlled trials with larger numbers of patients would be needed to increase the statistical power of the tests. The strength of the study is that we specifically evaluated the outcomes of PER therapy in childhood absence epilepsy, also in PER monotherapy, and that we provided data on tolerability through standardized neuropsychological tests.

## Conclusions

Considering that some forms do not respond to first choice antiseizure medications, and considering some limitations of use (e.g., contraindication to valproic acid in fertile women), it would be useful to evaluate the use of the new antiseizure medications in childhood absence epilepsy. The PER would appear to have a broad spectrum of action on both partial and generalized seizures. Our clinical experience in real life showed that PER appears to be effective in the control of absence seizures in childhood absence epilepsy, with a favorable tolerability profile. PER would seem effective on absence seizures even in monotherapy. Further studies with larger samples, longer follow-up and controlled vs. placebo or first choice antiseizure medications are needed to confirm these data.

## Data availability statement

The raw data supporting the conclusions of this article will be made available by the authors, without undue reservation.

## Ethics statement

The studies involving human participants were reviewed and approved by Campania Sud Ethics Committee. Written informed consent to participate in this study was provided by the participants' legal guardian/next of kin.

## Author contributions

Conceptualization and write original draft preparation: FO and GP. Methodology: RM, SA, and LA. Formal analysis: VV and GP. Data curation: VV, CS, CP, VS, RR, and GB. Writing, review and editing: GP, GS, LV, and SD. Supervision: FO and AO. All the authors have read and agreed to the published version of the manuscript.

## Conflict of interest

GS was employed by Azienda Sanitaria Locale Salerno. The remaining authors declare that the research was conducted in the absence of any commercial or financial relationships that could be construed as a potential conflict of interest.

## Publisher's note

All claims expressed in this article are solely those of the authors and do not necessarily represent those of their affiliated organizations, or those of the publisher, the editors and the reviewers. Any product that may be evaluated in this article, or claim that may be made by its manufacturer, is not guaranteed or endorsed by the publisher.
